# Draft Genome Sequence of a Novel Marine Anaerobic Ammonium-Oxidizing Bacterium, “*Candidatus* Scalindua sp.”

**DOI:** 10.1128/MRA.00297-19

**Published:** 2019-05-16

**Authors:** Muhammad Ali, Dario Rangel Shaw, Pascal E. Saikaly

**Affiliations:** a Biological and Environmental Science and Engineering Division, Water Desalination and Reuse Research Center, King Abdullah University of Science and Technology, Thuwal, Saudi Arabia; Indiana University, Bloomington

## Abstract

A novel anaerobic ammonium-oxidizing (anammox) bacterium was detected in an upflow column reactor treating synthetic nitrogen-rich saline solution. Here, we assembled a 4.59-Mb draft genome sequence of this bacterium, identified as a member of the genus “*Candidatus* Scalindua,” that has 84% nucleotide-level genomic similarity with the closest related anammox bacterium (“*Candidatus* Scalindua rubra”).

## ANNOUNCEMENT

Anaerobic ammonium-oxidizing (anammox) bacteria play a vital role in the global nitrogen cycle and in energy-efficient treatment of N-rich wastewaters (1). There are more than 100 full-scale anammox installations worldwide for the treatment of N-rich wastewaters (2), and there is now growing interest in applying anammox bacteria for the treatment of N-rich saline wastewaters (3). Thus, we started a 1-liter upflow column reactor (XK 50/60 column; GE Healthcare, UK) for the treatment of synthetic N-rich saline solution prepared using fresh Red Sea water (∼3.5% salinity) containing NH_4_^+^ and NO_2_^–^ (∼5 mM each). The reactor was operated at ambient temperature (35°C) with a constant feeding rate of 2.2 liters · day^−1^. The column reactor was inoculated with anammox biofilm attached to a nonwoven fabric sheet harvested from another reactor (4).

The biomass was extracted from the upflow column reactor under steady-state conditions, and genomic DNA was extracted using a DNA extraction kit (FastDNA Spin kit for soil; MP Biomedicals) according to the manufacturer’s instructions. The DNA was quantified using a Qubit fluorometer (Thermo Fisher Scientific, USA), and 50 ng was used to prepare Nextera libraries following the manufacturer’s instructions (Illumina, USA). The prepared DNA libraries were paired-end sequenced (2 × 250 bp) on a HiSeq 2500 instrument (Illumina) and generated approximately 44 million reads in total. The sequence reads were trimmed for Nextera adaptors using cutadapt v. 1.10 (5) with a minimum Phred score of 20 and a minimum length of 150 bp. The trimmed reads were assembled using SPAdes v. 3.7.1 (6). The reads were mapped back to the assembly using Burrows-Wheeler Aligner (BWA) v. 0.7.15-r1142-dirty (7) to generate coverage files for metagenomic binning. Open reading frames (ORFs) were predicted in the assembled scaffolds using Prodigal (8). A set of 117 hidden Markov models (HMMs) of essential single-copy genes were searched against the ORFs using HMMER3 (http://hmmer.janelia.org/) with default settings, with the exception that the option “-cut_tc” was used (9). Identified proteins were taxonomically classified using a BLASTP search against the RefSeq v.52 protein database with a maximum E value cutoff of 10^−5^. MEtaGenome ANalyzer (MEGAN) was used to extract class-level taxonomic assignments from the BLASTP output (10). The script network.pl (http://madsalbertsen.github.io/mmgenome/) was used to obtain paired-end read connections between scaffolds. The 16S rRNA genes were identified using BLAST v. 2.2.28+ (11) and were classified using SINA v. 1.2.11 (12) with the minimum identity adjusted to 0.80. The required data set for binning was generated according to the description in the mmgenome package v. 0.6.3 (13). The genome was extracted by using the mmgenome package in R v. 3.3.1 (14), and the extracted genome was annotated using PROKKA v. 1.12-beta (15).

A 4.59-Mb genome sequence comprising 121 contigs (GC content of 41% and *N*_50_ value of 92,628 bp) was obtained, and 2,565 gene-coding regions, 41 tRNAs, and a single rRNA (*rrn*) operon were annotated. The genome is 90% complete based on CheckM v1.0.5 (16). The calculated average nucleotide identity with the closest related anammox bacterium (“*Ca*. Scalindua rubra” [GenBank accession number MAYW00000000]) was 83.72%, and the genome therefore is considered novel.

### Data availability.

This whole-genome shotgun project has been deposited at DDBJ/ENA/GenBank under the accession number RBMW00000000. The Sequence Read Archive (SRA) accession number is SRR7904086. The BioProject accession number is PRJNA492998.
